# Traditional agroecosystems as conservatories and incubators of cultivated plant varietal diversity: the case of fig (*Ficus carica *L.) in Morocco

**DOI:** 10.1186/1471-2229-10-28

**Published:** 2010-02-18

**Authors:** Hafid Achtak, Mohammed Ater, Ahmed Oukabli, Sylvain Santoni, Finn Kjellberg, Bouchaib Khadari

**Affiliations:** 1 INRA, UMR 1098, Développement et Amélioration des Plantes (DAP), Bat. 3, Campus CIRAD TA A 96/03, Av. Agropolis, 34398 Montpellier Cedex 5, France; 2 Montpellier SupAgro, UMR 1098 DAP, Bat. 3, Campus CIRAD TA A 96/03, Av. Agropolis, 34398 Montpellier Cedex 5, France; 3 Faculté des Sciences de Tétouan, Diversité et Conservation des Systèmes Biologiques, BP 2062, M'hannech II Tétouan, Maroc; 4 INRA, UR Amélioration des Plantes et Conservation des Ressources Phytogénétiques, BP 578 Meknès, Maroc; 5 INRA, UMR 1097, Diversité et Adaptation des Plantes Cultivées (DiA-PC), Bat. 33, 2 place Viala, 34060 Montpellier Cedex 2, France; 6 CNRS, UMR 5175, Centre d'Ecologie Evolutive et Fonctionnelle (CEFE), 1919 route de Mende, 34293 Montpellier Cedex 5, France; 7 Conservatoire Botanique National Méditerranéen de Porquerolles, UMR 1098 DAP, 76 A, Av. Gambetta, 83400 Hyères, France

## Abstract

**Background:**

Traditional agroecosystems are known to host both large crop species diversity and high within crop genetic diversity. In a context of global change, this diversity may be needed to feed the world. Are these agroecosystems museums (i.e. large core collections) or cradles of diversity? We investigated this question for a clonally propagated plant, fig (*Ficus carica*), within its native range, in Morocco, but as far away as possible from supposed centers of domestication.

**Results:**

Fig varieties were locally numerous. They were found to be mainly highly local and corresponded to clones propagated vegetatively. Nevertheless these clones were often sufficiently old to have accumulated somatic mutations for selected traits (fig skin color) and at neutral loci (microsatellite markers). Further the pattern of spatial genetic structure was similar to the pattern expected in natural population for a mutation/drift/migration model at equilibrium, with homogeneous levels of local genetic diversity throughout Moroccan traditional agroecosystems.

**Conclusions:**

We conclude that traditional agroecosystems constitue active incubators of varietal diversity even for clonally propagated crop species, and even when varieties correspond to clones that are often old. As only female fig is cultivated, wild fig and cultivated fig probably constitute a single evolutionary unit within these traditional agroecosystems. Core collections, however useful, are museums and hence cannot serve the same functions as traditional agroecosystems.

## Background

High yield agriculture based on elite crop varieties and high inputs results in loss of both numbers of crop plants and genetic resources within crops, thus threatening crop biodiversity and the nutritional safety of humanity [1]. To preserve crop diversity, traditional landscapes may have to be preserved [2]. In analogy with the concept of "biodiversity hotspot" used to identify priority areas for the conservation of wild species [3], traditional agroecosystems could be considered as main conservatories of crop biodiversity [4]. Indeed in 2002 the FAO started an initiative for the conservation and adaptive management of Globally Important Agricultural Heritage Systems http://www.fao.org/nr/giahs/en/. Although they are quite diverse, these agroecosystems exhibit common features such as 1) a high diversity of crop species, 2) the use of diversified traditional varieties, 3) sustainable agriculture, 4) low inputs associated with traditional farming practices and 5) the farmers obtaining a sizable proportion of their seeds (or vegetative equivalents) from their own harvest [5]. For instance, a survey in continental oases in northern Oman recorded 107 different crop species belonging to 39 families, including 33 fruit species [6]. This large biodiversity was successfully achieved despite the constraints of a small scale cropping system under arid and semi-arid conditions. Similarly, a study of 27 crop species in traditional agroecosystems distributed in eight countries over the five continents [7] demonstrated that such agroecosystems maintain considerable within crop genetic diversity. Traditional agroecosystems are either the repositories of crop diversity, or the place where extant crop diversity was fostered. Hence investigating within crop species genetic diversity and its geographic variation would help understanding genetic resources and dynamic processes of past and present domestication and subsequent diversification.

The biodiversity hotspot concept is associated with a major pattern of biodiversity: it increases close to the equator, and decreases towards the poles [8]. Two main ideas have been suggested to explain this global biodiversity pattern. Equatorial regions are a museum of biodiversity preserving ancient biodiversity, and/or they are a cradle generating new biodiversity [9]. If agroecosystems are hosting huge crop biodiversity, should we consider them as museums or as incubators of crop biodiversity, or as both? For long term crop management policies and hence to feed the world, the answers to this question is of a great importance.

The Mediterranean basin is one of 25 hotspots of biodiversity in the world. It hosts 25,000 species, of which 13,000 are endemic, this later group representing 4.3% of the worldwide flora [3]. It is the largest biodiversity hotspot on earth (over 2,000,000 km^2^) and it includes several separate refuge areas [10]. Traditional agroecosystems are still found all over the Mediterranean region in mountains and oases. However several of these traditional agroecosystems may be of particular importance for preserving crop biodiversity. Indeed, many plant species were originally domesticated close to the Eastern shores of the Mediterranean. Hence, we might encounter contrasted patterns of genetic diversity within crops throughout the Mediterranean area, with more crop diversity available in the Eastern Mediterranean.

The process of domestication seems to have been diffuse, with prolonged cultivation of undomesticated forms, and prolonged genetic exchanges of domesticated forms with local wild relatives, at least for crops propagated by seeds [11,12]. With a such domestication process, traditional agroecosystems located in the East Mediterranean may be most important for preservation of crop genetic resources. In addition, the domestication process of clonally propagated crops, particularly fruit trees, is often thought to have been an instant or almost instant process [13,14] building on the idea that genotypes presenting the whole suite of agronomic traits of interest may have arisen by chance within totally natural populations [15]. This may qualify as a silver bullet hypothesis. If we follow this hypothesis, domestication was instantaneous, and followed by subsequent clonal propagation. Hence we would expect that extant varieties are old, probably limited in number, and that they represent the gene pool of the original region of domestication. The wild progenitors of some of these clonal crops still grow all around the Mediterranean region. This is true for three most symbolic crops in these regions such as olive, grape wine and fig. Therefore, we may ask, within such species, whether extant varietal genetic diversity in traditional agroecosystems reflects the propagation of old widespread clones, or old local clones, or recent local clones. We may even ask whether varieties could be fuzzy aggregations of genotypes (landraces) [16].

We chose to address this question in fig which presents us with a particularly fascinating situation as it is extremely easy propagated via cuttings, and was domesticated extremely early in the Near East, contemporarily with cereal crops, 9-12,000 BP [17]. Fig, *Ficus carica *L., is dioecious. Female trees produce the edible crop. Male trees produce pollen and their figs host the pollinator, *Blastophaga psenes *[18]. Each fig variety is a clone of female tree that are propagated through cuttings. Some fig varieties may produce seedless fig fruits without pollination while other varieties require pollination for successful fruit set [19]. Female figs produce seeds if pollinated. Male figs are often collected far from zones of fig cultivation and suspended in cultivated female trees to ensure pollination [20].

Phylogeographic studies based on cytoplasmic genes showed that wild fig was present all over the Mediterranean basin before domestication [21]. We investigated the genetic diversity of fig varieties in Moroccan traditional agroecosystems. Morocco is at the Western limit of the natural range of fig, as far away as possible (over 3500 km) from postulated places of domestication. Hence, if domestication begun and ended in the Eastern Mediterranean, then we expect to observe limited diversity so far away from the original zone of domestication. We also expect to observe lack of spatial genetic structure within Morocco, or simply a decrease of diversity when further away from the shores of the Mediterranean.

We made extensive collections of fig cultivars *in situ*, in order to 1) test whether cultivars are effectively highly local, 2) detect whether some of these cultivars are old, and 3) establish what insights into the history of fig cultivation could be drawn from extant genetic diversity and its spatial structuring.

We show here that in traditional agroecosystems, fig varieties are true clones, highly diversified, often highly local. Nevertheless they are often sufficiently old to have accumulated somatic mutations. Spatial genetic structure resembles what would be expected for a wild plant at mutation/drift/migration equilibrium. We conclude that the Moroccan traditional agroecosystems are at the same time museums and incubators of fig variety diversity, in a dynamic system preserving old, local varieties and generating new ones locally.

## Results and Discussion

**Figure 1:**
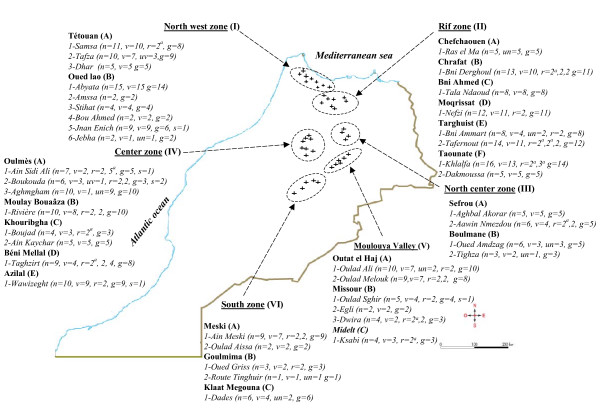
**Sampling locations and fig sample diversity**. Six geographic zones were defined I, II, III, IV, V and VI. Letters A, B, C, D, E and F correspond to subzones, and within each subzone, sites are indicated. v = number of sampled local variety names; un = number of sampled unnamed variety; for varieties sampled several times in a site, r is the number of repeats of each local variety name (r = 2, 3, 5 means that 3 varieties have been sampled several times, one 2 times, the second 3 times and the third 5 times); g = number of genotypes sampled; s = number of varieties presenting somatic mutations for fig skin color; a = fig trees under the same variety name and genotype.

277 cultivated trees were sampled throughout traditional Moroccan agroecosystems distributed over 40 sites that we grouped into 6 geographical zones (Figure 1). During field collection, we noted that, within each site, trees designated by the same name (local variety) shared highly similar morphological traits. To maximize genetic diversity of our sampling we generally collected a single individual per variety per site. Nevertheless, in a number of cases we sampled twice the same local variety within a site or within adjacent sites. Such samples systematically shared a same genotype. Hence genetic evidence confirms the obvious conclusion from phenotypic observation that local varieties are generally clones.

### SSR polymorphism and its discrimination power

The 277 individuals genotyped were separated into 194 distinct molecular profiles using 17 SSR loci (see Additional File 1). Genetic parameters for each locus are given in Table 1[22-25]. Overall, observed heterozygosity was higher than expected heterozygosity. The discriminating power per locus, *D*_*i *_(probability of distinguishing two randomly chosen clones), ranged from 0.495 (LMFC26) to 0.979 (LMFC30) with a mean of 0.70 (Table 1). Hence the probability of confusing a randomly chosen clone with another one (under the hypothesis of statistical independence of the loci) was *Π*(1-*D*_*i*_) = 5 × 10^-11^. With only 38,226 pairwise comparisons (including identical genotypes) in our data set, all cases of genotype identity should correspond to clones.

**Table 1:** Genetic parameters of the 17 SSR loci used in this study.

Locus	*A*	Size range (in bp)	*H*_*O*_	*H*_*E*_	*F*_*IS*_	*D*
MFC1^a^	5	161-195	0.620	0.629	0.016	0.841
MFC2^a^	7	156-190	0.599	0.602	0.008	0.880
MFC3^a^	9	96-136	0.818	0.760	-0.074	0.851
MFC4^a^	5	216-226	0.524	0.493	-0.060	0.652
MFC8^b^	2	173-177	0.508	0.490	-0.033	0.619
MFC9^b^	7	188-211	0.636	0.582	-0.090	0.786
MFC11^b^	7	181-203	0.604	0.569	-0.059	0.585
MFC12^b^	4	152-167	0.578	0.552	-0.045	0.743
FSYC01^c^	5	117-160	0.455	0.451	-0.005	0.842
FSYC04^c^	2	181-183	0.529	0.502	-0.053	0.595
LMFC19^d^	8	296-312	0.433	0.398	-0.086	0.573
LMFC24^d^	4	272-278	0.460	0.456	-0.006	0.646
LMFC26^d^	3	224-236	0.235	0.223	-0.051	0.495
LMFC28^d^	5	192-203	0.562	0.558	-0.004	0.733
LMFC30^d^	11	231-261	0.904	0.820	-0.100	0.979
LMFC32^d^	9	197-225	0.433	0.415	-0.039	0.628
LMFC34^d^	2	245-247	0.492	0.486	-0.009	0.519

*A*: number of alleles, *H*_*O*_: observed heterozygosity, *H*_*E*_: expected heterozygosity, *F*_*IS*_: within population fixation index, *D*: discriminating power. Primers developed by: ^a ^Khadari et *al*. [22], ^b ^Achtak et *al*. [23], ^c ^Ahmed et *al*. [24] and ^d ^Giraldo et *al*. [25].

**Figure 2:**
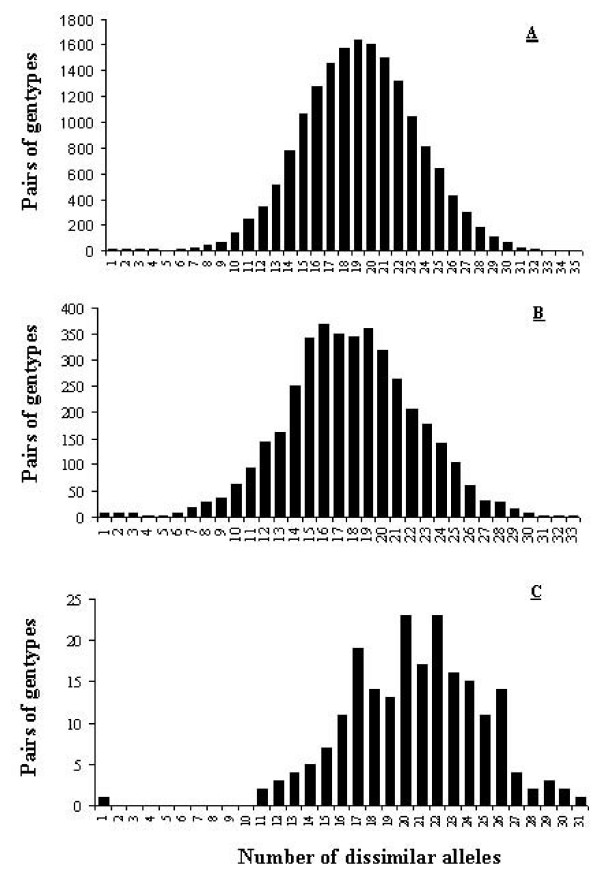
**Frequency distribution of genetic dissimilarity for all pairwise comparisons between cultivated fig genotypes**. (A) complete data set; (B) in mountain agroecosystem; (C) in oasis agroecosystem. Genetic differences among genotypes are retained in the oasis agroecosystem, despite the low number of genotypes cultivated (21). Note on the three graphs the bimodal shape of the curve with a very small peak for differences of 1-3 alleles.

We plotted the distribution of number of allelic differences between the 194 different genotypes in order to visualize the distribution of genetic differences between genotypes, (Figure 2A; 18,721 pairwise comparisons, excluding identical genotypes). The distribution ranged from 1 to 34 differences, presented a major peak at 19-20 differences and a very distinct, but very small, peak at 1-3 differences. The probability to observe by chance two or more genotypes that were distinguished by 3 alleles was 2.6 × 10^-6^. Further, individuals whose genotypes were identical or differed by only 1-3 alleles were morphologically highly similar (see Additional File 2). The systematic association of genetic similitude for neutral markers with morphological similarity allows to conclude that all these trees belonged to a single original clone and that some had accumulated somatic mutations. Further, the shape of the pairwise genetic difference curve suggests that, beyond the case of the few genotypes deriving from each other by somatic mutations, all other genotypes are the product of sexual reproduction. We chose to be highly conservative in our estimate of which genotypes represented somatic mutations. Indeed the curve suggests that the limit may be better placed above 6 differences and indeed the probability of observing by chance two genotypes differing only by 6 alleles was still low, at 0.0017.

**Figure 3:**
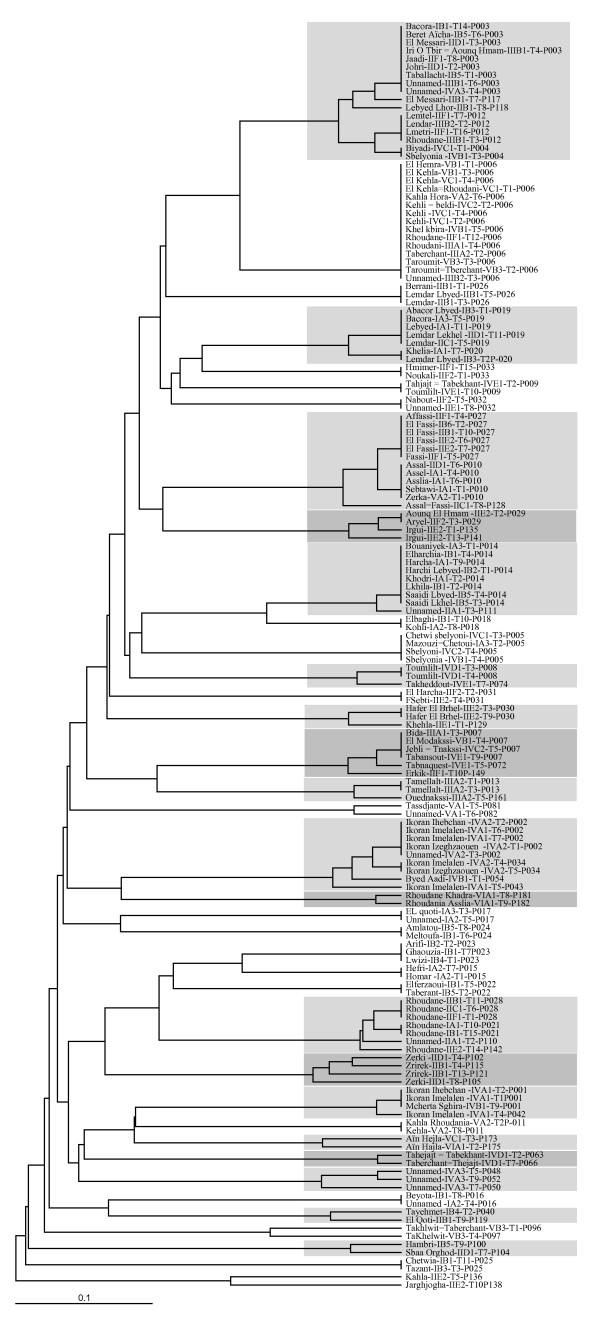
**Genetic similitude among fig varieties**. Samples grouped within a box correspond to highly similar genotypes that most probably derive from each other by somatic mutation. These similar genotypes often bear similar variety names. After the variety name, the roman number indicates zone of sampling, the letter the subzone, followed by a number giving the precise site of sampling, Tx indicates the tree number × within site and Pxxx indicates genotype number xxx (see Additional File 1).

Hence, we classified the 194 genotypes into 152 genotype groups (clones) separated by at most 3 alleles, which were distinguished from all other genotypes by 4 to 34 alleles. Out of these groups of genotypes, 128 contained a single individual while 24 groups contained more than one individual and represented collectively 66 genotypes. Often a variety name was found to be associated with the same clone (identical or almost identical genotype) in different sites, conforting our conclusions. Numbers of trees sharing the same genotypes are given in Additional File 3, while Additional File 4 and Figure 3 provide a series of cases of genotypes differentiated by 1-3 alleles and sharing the same variety name. While we have no data on mutation rates in somatic lines, the presence of such mutations within clonal lineages suggests that these varieties are old.

### Variety names and characterization

Out of 277 sampled fig trees, 246 were named by the local farmers while for 31 fig trees, the interviewed farmers did not provide any name (see Additional File 1). These 31 unnamed trees corresponded to 30 genotypes out of which four corresponded genetically and morphologically to known varieties ('Ikoran Imelalen', 'El Messari', 'El kehla' and 'Beyota') and three were very similar genetically and morphologically to the 'Saaidi' and 'Rhoudane' varieties. The remaining 23 genotypes were distinct from previously defined varieties.

Synonymy was observed for 23 genotypes, with 2 to 7 denominations per genotype (Figure 3, Additional File 3). Two situations were observed. True synonymy was observed when the different fig trees presented identical pomological traits such as the varieties 'Johri' and 'El Messari' (green fig skin color, flattened pyriform fruit shape and red internal color). This situation was encountered for 20 genotypes. False synonymy was observed for fig trees known under the same generic denomination to which a descriptor of fig skin color was added. In these cases the leaves and the figs presented similar morphologies but fruit color was different. Six instances of the latter situation were encountered (Figure 3, Additional File 3). They included for instance 'Saaidi Lbyed-IB5-T4-P014' (white skin color) and 'Saaidi Lkhel-IB5-T3-P014' (black) in the North west zone, 'Ikoran Ihebchan-IVA2-T2-P002' (black) and 'Ikoran Imelalen-IVA1-T1-P001' (white) in the Center zone (Figures 1 and 3).

We suggest that the second type of synonymy corresponds to cases of somatic mutations. A similar situation has previously been reported in Cataluña for 'Col de Dame blanche', 'Col de Dame grise' and 'Col de Dame noire' which are genetically and morphologically identical and only differ by skin color [26] and in Slovenia for 'Green Matalon' and 'Black Matalon' [27]. Such mutations have been reported in *Vitis vinifera *[28], and indeed, in Brazil, a single wine producer successfully selected 2 clonal color variants [29]. In our study, each time we encountered several color forms within a variety, they occurred within the same zone, but not necessarily within the same site (see Figure 1 and Additional File 3). This suggests that varieties have a prolonged local history.

We grouped several variety names as highly similar because they had the same meaning albeit in different languages or dialects (see Additional File 4). For instance, the names 'Ikoran Ihebchan -IVA1-T3P041', 'Kahla-VIA1-T4P177', 'Kohli-IA2-T8P018', and 'Taberchante-VA1-T1-P077' sampled in the central region, in the oases, in the North west and in the Moulouya valley, respectively, all corresponded to black figs presenting turbinate fruit shape, but their genotypes were distinctive. Thus cases of homonymy involved 31 distinct denominations corresponding to 181 fig trees and 147 genotypes (see Additional File 4). In a number of cases such homonymy corresponded to highly similar genotypes. Nevertheless, the denominations representing most cases of homonymy were referring to fruit color. Denominations referring to White, Black and Green color represent a total of 55 genotypes, i.e. 1/3 of the 164 genotypes sampled with variety denomination.

Depending on the genetic relationships between genotypes, three types of homonymy were distinguished (see Additional File 4). First we observed homonymy between highly similar genotypes (= within a clone) such as within the varieties 'Rhoudane', 'Zerki' and 'Byed', which included respectively three, four, and four very closely related genotypes. As stated above these correspond most probably to cases of somatic mutation within clone, and do not really constitute cases of homonymy. Second we observed cases of homonymy grouping varieties presenting similar pomological traits but clearly distinct genotypes, such as the cultivars 'Aïn Hajla', 'Rhoudane', 'Kehla' and 'Biyadi', representing respectively two, six, eight, nine and six distinct genotypes. Finally we observed cases of homonymy grouping varieties presenting different pomological traits and different genotypes (six cases; Additional File 4).

Only eight clones were present in several geographic zones. This was the case for instance for 'Assel-IA1-T4-P010' (North west zone), 'Assal-IID1-T6-P010' (Rif zone), 'Zerka-VA2-T1-P010' (Moulouya valley). These eight non local clones corresponded to widely known varieties, such as 'Assal-IID1-T6-P010' = 'Sebtawi-IA1-T1-P010' = 'Zerka-VA2-T1-P010'; 'Rhoudane-IIF1-T12-P006' = 'Rhoudani-IIIA1-T4-P006' = 'El Kehla (Rhoudani)-VC1-T1-P006' and 'Bacora-IA3-T5-P019' = 'Lemdar-IIC1-T5-P019' (see Additional File 3). Hence, in Morocco, most fig varieties are cultivated over a limited spatial. Concurrently, within a geographical zone, varieties often correspond to a single specific clone. For instance, in the Rif, the 81 trees analyzed were assigned to 43 named varieties (and 7 unnamed) and corresponded to 64 genotypes (grouped into 35 clones when including within a clones all genotypes that differed by at most three alleles).

Hence in traditional Moroccan agroecosystems fig local varieties are clones and they are generally highly local and diversified (on average 8 local varieties were collected per site in the Rif region). At least some of these local varieties were sufficiently old to have accumulated somatic mutations on neutral genetic markers and on selected traits.

### Genetic diversity within and among geographical groups

Similar numbers of alleles were observed within each geographic zone, except the North center zone which presented fewer varieties, few local genotypes and as a consequence fewer alleles (Table 2). Surprisingly in the South zone, all genotypes were local and allele diversity was similar to that observed in other zones. Among the 95 observed alleles, three were exclusively detected in the center zone (MFC3-133, LMFC30-259, LMFC28-192), two in the Moulouya valley (MFC9-188, LMFC24-278), two in the North west zone (LMFC19-306, LMFC32-225) and four in the South zone (MFC3-96, MFC2-190, MFC9-211, LMFC30-243). Expected heterozygosity was highest in the South zone (0.558) and lowest in the Rif zone (0.495).

**Table 2:** Genetic diversity within geographical zone.

Geographic zone		trees analyzed	named varieties	unnamed varieties	genotypes	local genotypes	*N*	*N*_*A*_	*H*_*E*_	*H*_*O*_	*F*_*IS*_	p-value
North west (I)		60	43	4	36	31	66	3.88	0.523	0.559	-0.0699	0.0187
Rif (II)		81	43	7	64	58	70	4.12	0.495	0.540	-0.0968	0.0003
**Mountain agroecosystems **^a^		**141**	**76**	**11**	**96**	**89**	**77**	**4.53**	**0.510**	**0.548**	**-0.0724**	**0.0005**
North center (III)		20	12	4	15	12	54	3.18	0.533	0.558	-0.0502	0.1477
Center (IV)		61	23	10	45	40	70	4.12	0.511	0.557	-0.0904	0.0156
Moulouya valley (V)		34	19	2	27	24	70	4.12	0.518	0.507	0.0219	0.4292
South (VI)		21	15	3	21	21	72	4.24	0.558	0.571	-0.0242	0.2898
**Oasis agroecosystems **^b^		**21**	**14**	**3**	**21**	**21**	**72**	**4.24**	**0.558**	**0.571**	**-0.0242**	**0.2898**

*N*: total number of alleles observed within each zone; *N*_*A*_: mean number of alleles per locus; *H*_*O*_: observed heterozygosity; *H*_*E*_: expected heterozygosity; *F*_*IS*_: intrapopulation fixation index. ^a ^Moutain agroecosystems (= Rif and North west zones); ^b ^Oasis agroecosystems (= oases of the South Morocco).

There is no published data available on fig genetic diversity in traditional agroecosystems based on a sufficient number of genetic markers to discriminate clones. However, ongoing work in Lebanon and in the Tizi Ouzou area (Algeria) using the same markers (Chalak, **pers**. comm.; Daoudi, **pers**. comm.) suggest the presence of similar level of diversity as in Northern Morocco. These areas correspond to traditional agroecosystems mainly based on subsistence agriculture, with orchards presenting several fruit species grown together and several varieties per species [30,31]. Hence, the pattern observed for fig variety diversity in Morocco can probably be transcribed to most traditional agroecosystems around the Mediterranean. How the pattern may shift outside the range of wild *Ficus carica *remains an open question.

**Figure 4:**
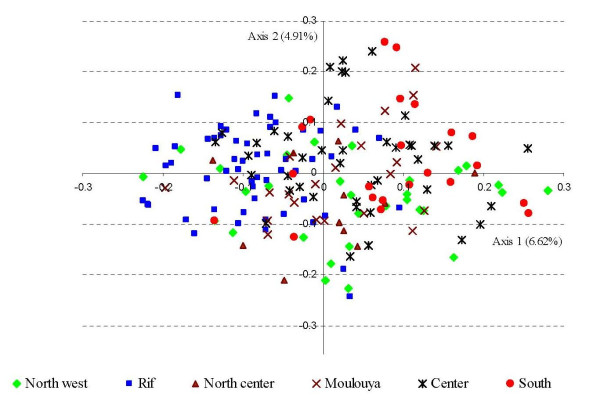
**Separation of genotypes according to zone of origin on the two first axes of the Factorial Correspondence Analysis**. The Southern zone (in red) and the Rif zone are separated (in blue).

**Figure 5:**
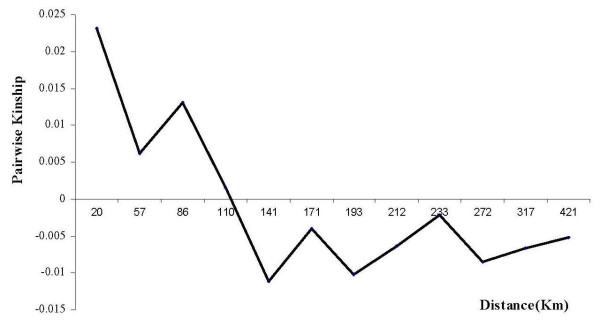
**Pairwise kinship coefficient between genotypes as a function of geographic distance**.

Genetic differentiation among the six geographic zones was about 4% (*F*_*ST *_= 0.038). Pairwise comparisons showed contrasted *F*_*ST *_values ranging from 0.017 to 0.068 (Table 3). The highest differentiation (*F*_*ST *_= 0.07) was noted between the Southern zone and the Rif zone. These two zones were also the sole zones clearly separated on the two first coordinate axes of the Factorial Correspondence Analysis (Figure 4). A significant spatial genetic structure was observed (p < 10^-6^). Pairwise Loiselle kinship coefficients decreased significantly with distance (Figure 5), and were more strongly correlated with log than with linear distance, whatever the range of distances incorporated in the calculus. Such a pattern would be interpreted in natural populations as isolation by distance with no rupture in gene flow [32].

**Table 3:** Pairwise *F*_*ST *_values between samples from the different geographic zones.

	North west	Rif	North center	Center	Moulouya
Rif	0.028**				
North center	0.026*	0.046**			
Center	0.021***	0.030***	0.025*		
Moulouya	0.027***	0.031**	0.026*	0.017**	
South	0.038***	0.068***	0.042*	0.018*	0.029**

* p < 5.10^-3^, ** p < 10^-6^, *** p < 10^-9^

We may reconcile the three sets of analyses (FCA, *F*_*ST *_and pairwise Loiselle kinship coefficients) by suggesting that we have here the image of spatial genetic structure as could be expected in natural populations for a situation of mutation/migration/drift processes at equilibrium resulting in some geographic variation in genetic background without geographic variation in genetic diversity. Within this global pattern, the North west zone appears to be slightly atypical, a feature which could have been predicted. Indeed, the region is the most affected by neighboring cities and as such represents a less traditional agroecosystem, slightly blurring the picture.

The pattern of isolation by distance, with no clines in diversity, is a signature of a genetic equilibrium situation, with no trace of a past colonization process. This feature and the quasi-absence of widespread varieties, is suggestive of a cultivation system based on varieties that originated locally, mainly from the local gene pool.

### Varietal and genotypic diversity in mountain and oasis agroecosystems

Quite interestingly, traditional mountain agroecosystems (North west and Rif zones) presented much more varietal diversity than traditional oasis agroecosystems (South zone) (Table 2, Figure 2B and 2C). However they presented almost identical numbers of alleles. This result was obtained despite our sampling only 21 trees in the oases against 141 trees in the North west and Rif zone. This suggests that fig varietal and genetic diversity available in oases is threatened, maybe due to their small surface, while the one available in the mountain agroecosystems will be more resilient.

## Conclusions

Traditional Moroccan agroecosystems contain substantial fig varietal and genetic diversity. While fig varieties are true clones and not landraces [16], the distribution of differences between genotypes shows that this diversity arose through sexual reproduction and only marginally, through somatic mutation. Hence the silver bullet hypothesis of instantaneous domestication of clonal plants [13] does not apply, at least today, to fig. In that perspective fig is similar to other clonally propagated plants from other parts of the world for which sexual reproduction has been important and often still is. Such species include for instance Cassava [33] and Agave [34] in America or Enset [35] in Africa. Further, in fig, sexual production of new varieties almost obligatorily involves crosses with wild figs. Indeed, it is a dioecious species, and male figs used for pollination are collected on any tree in the neighborhood, and when male figs are cultivated within a village, their potential genetic qualities for siring agronomically interesting crops is not taken into account. Preliminary data from the Rif zone confirms close genetic relationship between local varieties and wild growing fig trees. As such fig cultivation in its native range fits the global picture of frequent hybridization of cultivated plants with their wild relative [36].

However the case of fig is particular as new varieties must (almost) systematically result in the incorporation of hybrids between wild and cultivated plants. We may thus suspect that in all traditional Mediterranean agroecosystems located within fig natural habitat, cultivated figs and wild growing figs locally form a single evolutionary unit. Hence such traditional agroecosystems are effectively incubators of fig variety diversity in a dynamic incorporating wild growing as well as cultivated trees. This is not always the case in clonally propagated plants. For instance, while sexual reproduction seems to be most important in traditional Cassava cultivation, genetics allow to trace its origin to a single region of the range of its progenitor, *Manihot flabellatus *[37]. The domestication process of monoecious and dioecious plants may turn out to be quite different.

In a context of ongoing rapid climatic change, the nutritional quality, and toxicity of crops may change dramatically [38]. A dynamic management of genetic resources as observed here in traditional agroecosystems may prove essential for responding to such new challenges.

## Methods

### Fig sampling

Traditional agroecosystems are still present in Morocco, in the Rif and Atlas mountains in Northern and central areas and in oases in the South east. A survey in the Rif agroecosystems showed that 28 crop species were cultivated including 14 fruit species [31]. A high diversity of fruit crops was also observed in the South Moroccan oases.

Field trips to collect plant material covered all territories of Morocco presenting traditional agroecosystems (Figure 1). They were done in June and August-September in order to observe first or second crop figs, respectively (fig varieties produce either both first crop and second crop or only the second crop). Collections were made in 2005 and 2006. This allowed characterizing the different varieties and establishing their geographical range. Field observations and some genetic data (Achtak et *al*. unpublished) had shown that within the range of each prospection site or village, each variety corresponded generally to a single genetic clone. The sampling strategy could therefore be focused on diversity, using pomological observation following the IPGRI recommendation [39] and interviews with farmers. Thus, for each prospection site, we sampled one individual of each of the cultivated varieties. When we had a doubt on the perfect identity of vegetative and pomological traits within a variety within a site, or when a farmer suggested that there were two types within a variety, then we collected both forms. Hence genetic homogeneity within variety was assessed within site when there was any hint of a doubt, and systematically, among sites. Local variety names were noted as given by farmers; photographs and GPS coordinates were recorded as references for each collected fig tree (see Additional File 1). The photographs allowed confronting a posteriori genotypic identity with morphological similitude. Six major geographical zones were surveyed (North west, Rif, North center, Center, Moulouya valley and South; Figure 1) and 277 trees representing 119 denominations were sampled.

### DNA extraction and SSR genotyping

Total genomic DNA was extracted from 200 mg of fresh young leaves of the 277 sampled fig trees using the DNeasy Plant Mini Kit (QIAGEN) according to the supplier's instructions with the following modification: 1% of Polyvinylpyrrolidone (PVP 40,000) was added to the buffer AP1.

We selected 17 loci among the developed SSR markers [22-25] based on their polymorphism and ease of scoring following the screening of 16 distinct Mediterranean varieties.

Microsatellite amplifications were performed according to the protocol described by Khadari et *al*. [40]. SSR genotyping was conducted in an automated capillary sequencer (ABI prism 3130 XL). Analyses were performed using the GENEMAPPER V3.7 software.

### Data analysis

For each SSR locus, alleles were detected and identified by locus name and allele size in bp. Genetic distances between fig genotypes were estimated according to the Jaccard similarity coefficient and UPGMA algorithm using a program developed by J. Brzustowski http://www.biology.ualberta.ca/jbrzusto/cluster.php. The corresponding phenogram was drawn based on the software Treeview 6.1. Discriminating power, D, was calculated for each SSR locus as *D*_*j *_= ∑*p*_*i *_[(*Np*_*i*_-1)/*N*-1)] [41] where *p*_*i *_was the frequency of the *i*-th molecular pattern revealed by locus *j*, and *N *was the number of genotypes. We used the *Dj *values to compute the exact probabilities of getting at least one pair of genotypes differing only at 0, 1, 2, 3, 4, 5 and 6 loci.

The number of alleles per locus (*A*), observed heterozygosity (*H*_*O*_), expected heterozygosity (*H*_*E*_) and Wright's fixation index (*F *= 1- *H*_*O*_/*H*_*E*_) were computed using the software Genetix 4.5 [42]. Genetic diversity was compared among geographic zones using parameters corrected for sample size [43]. Genetic differentiation between populations was assessed using *F*_*ST *_values and the software Genepop 3.1 [44]. The significance of population differentiation was estimated using exact tests [45].

To assess genetic isolation by distance, spatial genetic structure was investigated using a spatial autocorrelation method. Genetic relationships between all pairs of genotypes were regressed on the linear and the logarithmic geographical distance using the software SPAGeDi [46]. The kinship coefficient of Loiselle *et al*. [47], robust against the presence of low frequency alleles, was used. Significance of the regression coefficients was assessed through 10,000 permutations.

## List of Abbreviations

BP: Before Present; pers. comm.: personal communication; DNA: Deoxyribonucleic acid; FCA: Factor Correspondence Analysis; GPS: Global Positioning System; IPGRI: International Plant Genetic Resources Institute; pb: base pair; PCR: Polymerase Chain Reaction; PVP:Polyvinylpyrrolidone; SSR: Simple Sequence Repeat; UPGMA: Unweighted Pair Group Method with Arithmetic mean.

## Authors' contributions

BK designed and coordinated the study. HA, MA, AO and BK performed fig sampling. HA, SS and BK carried out the molecular analysis. HA, FK and BK performed the statistical analysis and wrote the first draft of the manuscript. All authors participated in the draft finalization and approved the final manuscript.

## Authors' information

HA is a geneticist who defended the PhD thesis entitled "domestication and diversification of fig, *Ficus carica *L., varieties in Morocco" in December 2009. MA is a Professor at University of Tétouan (Morocco), plant ecologist and working on agrobiodiversity. AO is a scientist at the Moroccan Agronomic Research Institute in charge of the management of fruit genetic resources. SS is an engineer, molecular biologist, in charge of the development and use of molecular markers. FK is a scientist in evolutionary biology particularly on biotic interactions, specialised on the *Ficus *pollinating wasps co-evolution. BK is a geneticist working within the conservation biology field and managing a Mediterranean fruit domestication and diversification program.

## Supplementary Material

Additional file 1 **List of the studied fig trees**. This table provides the list of studied fig trees with indications on their sampled geographic zone, sub-zone, site, name, SSR profile and the GPS coordinates. Click here for file

Additional file 2 **List of groups of closely related genotypes with skin color fruit**. This file describes a list of groups of closely related genotypes differed only by 1 to 3 alleles and considered to be somatic variants of a single clone. Click here for file

Additional file 3 **Cases of synonymy**. This file describes the cases of synonymy (several variety names for one genotype) observed among cultivated fig trees in Morocco. Click here for file

Additional file 4 **Cases of homonymy**. This file describes the cases of homonymy (several genotypes for one variety name) observed among cultivated fig trees in Morocco. Click here for file

## References

[B1] Esquinas-Alcazar J Protecting crop genetic diversity for food security: political, ethical and technical challenges Nature Reviews Genetics 2005 6 946 953 10.1038/nrg1729 16341075

[B2] Mooney H Cropper A Reid W Confronting the human dilemma: How can ecosystems provide sustainable services to benefit society? Nature 2005 434 561 562 10.1038/434561a 15800597

[B3] Myers N Mittermeier RA Mittermeier CG Da Fonseca GAB Kent J Biodiversity hotspots for conservation priorities Nature 2000 403 853 858 10.1038/35002501 10706275

[B4] Brush SB In situ conservation of landraces in centers of crop diversity Crop Science 1995 35 346 354

[B5] Zhu Y Chen H Fan J Wang Y Li Y Chen J Fan J Yang S Hu L Leung H Mew TW Teng PS Wang Z Mundt CC Genetic diversity and disease control in rice Nature 2000 406 718 722 10.1038/35021046 10963595

[B6] Gebauer J Luedeling E Hammer K Nagieb M Buerkert A Mountain oases in northern Oman: An environment for evolution and in situ conservation of plant genetic resources Genetic resources and crop evolution 2007 54 465 481 10.1007/s10722-006-9205-2

[B7] Jarvis D Brown AHD Cuong PH Panduro CL Moreno LL Gyawali S Tanto T Sawadogo M Mar I Sadiki M Hue NTN Reyes AL Balma D Bajracharya J Castillo F Rijal D Belqadi L Rana R Seddik S Ouedraogo J Zangre R Rhrib K Chavez JL Schoen D Sthapit B De Santis P Fadda C Hodgking T A global perspective of the richness and evenness of traditional crop-variety diversity maintained by farming communities PNAS 2008 105 5326 5331 10.1073/pnas.0800607105 18362337 PMC2291090

[B8] Hillebrand H On the generality of the latitudinal diversity gradient American Naturalist 2004 163 192 211 10.1086/381004 14970922

[B9] Mittelbach GG Schemske DW Cornell HV Allen AP Brown JM Bush MB Harrison SP Hurlbert AH Knowlton N Lessios HA McCain CM McCune AR McDade LA McPeek MA Near TJ Price TD Ricklefs RE Roy K Sax DF Schluter D Sobel JM Turelli M Evolution and the latitudinal diversity gradient: speciation, extinction and biogeography Ecology Letters 2007 10 315 331 10.1111/j.1461-0248.2007.01020.x 17355570

[B10] Médail F Quézel P Biodiversity hotspots in the Mediterranean basin: Setting global conservation priorities Conservation Biology 1999 13 1510 1513 10.1046/j.1523-1739.1999.98467.x

[B11] Willcox G Buxo R Herveux L Late Pleistocene and early Holocene climate and the beginnings of cultivation in northern Syria Holocene 2009 19 151 158 10.1177/0959683608098961

[B12] Weiss E Kislev ME Hartmann A Autonomous cultivation before domestication Science 2006 312 1608 1610 10.1126/science.1127235 16778044

[B13] Zohary D Unconscious selection and the evolution of domesticated plants Economic Botany 2004 58 5 10 10.1663/0013-0001(2004)058[0005:USATEO]2.0.CO;2

[B14] Kislev ME Hartmann A Bar-Yosef O Response to comment on "Early domesticated fig in the Jordan valley" Science 2006 314 1683 10.1126/science.1133748 16741119

[B15] Lev-Yadun S Ne'eman G Abbo S Flaishman MA Comment on "Early domesticated fig in the Jordan valley" Science 2006 314 1683 10.1126/science.1132636 17170278

[B16] Harlan JR *Crops and Man* American Society of Agronomy, Madison, WI 1975 1

[B17] Kislev ME Hartmann A Bar-Yosef O Early domesticated fig in the Jordan Valley Science 2006 312 1372 1374 10.1126/science.1125910 16741119

[B18] Kjellberg F Gouyon PH Ibrahim M Raymond M Valdeyron G The stability of the symbiosis between dioecious figs and their pollinator: a study of *Ficus carica *L. and *Blastophaga psenes *L Evolution 1987 41 693 704 10.2307/2408881 28564365

[B19] Condit IJ Fig varieties: a monograph Hilgardia, Berkeley 1955 23 323 538

[B20] Aumeeruddy-Thomas Y Hmimsa Y Ater M Khadari B Chevalier A, et al Beyond the divide between wild and domestic: domesticity, spatiality and practices pertaining to fig (*Ficus carica*) and olive (*Olea europaea*) agroecosystems in Morocco Crops and people: choices and diversity through time Oxfam, London in press

[B21] Khadari B Grout C Santoni S Kjellberg F Contrasting genetic diversity and differentiation among Mediterranean populations of *Ficus carica *L.: a study using mtDNA RFLP Genetic resources and crop evolution 2005 52 97 109 10.1007/s10722-005-0290-4

[B22] Khadari B Hochu I Santoni S Kjellberg F Identification and characterisation of microsatellite loci in the common fig (*Ficus carica *L.) and representative species of genus *Ficus* Molecular Ecology Notes 2001 1 191 193 10.1046/j.1471-8278.2001.00072.x

[B23] Achtak H Oukabli A Ater M Santoni S Kjellberg F Khadari B Microsatellite markers as reliable tools for fig cultivar identification Journal of American Society of Horticultural Science 2009 134 624 631

[B24] Ahmed S Dawson DA Compton SG Gilmartin PM Characterization of microsatellite loci in the African fig *Ficus sycomorus *L. (*Moraceae*) Molecular Ecology Notes 2007 7 1175 1177 10.1111/j.1471-8286.2007.01822.x

[B25] Giraldo E Viruel MA López-Corrales M Hormaza JI Characterization and cross-species transferability of microsatellites in the common fig (*Ficus carica *L.) Journal of Horticultural Science and Biotechnology 2005 80 217 224

[B26] Khadari B Lashermes P Kjellberg F RAPD fingerprints for identification and genetic characterization of fig (*Ficus carica *L.) genotypes Journal of Genetics and Breeding 1995 49 77 86

[B27] Bandelj D Jakse J Javornik B Bandelj D, Miklavcic MB, Vrhovnik I Development of microsatellite markers for identification of fig varieties in Istria The common fig (Ficus carica L.) in Istria (morphological, molecular and some chemical characteristics) 2008 Koper, Slovenia: Annales Publishing House 84 89

[B28] Yakushiji H Kobayashi S Goto-Yamamoto N Tae Jeong S Sueta T Mitani N Azuma A A skin color mutation of grapevine, from black-skinned Pinot Noir to white-skinned Pinot Blanc, Is caused by deletion of the functional *VvmybA1 *allele Bioscience, Biotechnology, and Biochemistry 2006 70 1506 1508 10.1271/bbb.50647 16794336

[B29] De Oliveira-Collet SA Zequim-Maia SH Mangolin CA de Fátima Pires da Silva Machado M Mutações e Recombinações Genéticas Pesquisa geram uvas coloridas Biotecnologia Ciência & Desenvolvimento 2005 35 28 35

[B30] Taiqui L Cantarino CM Elément historiques d'analyse écologique des paysages montagneux du Rif Occidental (Maroc) Mediterranea Serie de estudios biologicos 1997 16 23 36

[B31] Hmimsa Y Ater M Agrodiversity in the traditional agrosystems of the Rif mountains (North of Morocco) Biodiversity 2008 9 78 81

[B32] Vekemans X Hardy OJ New insights from fine-scale spatial genetic structure analyses in plant populations Molecular Ecology 2004 13 921 935 10.1046/j.1365-294X.2004.02076.x 15012766

[B33] Rival L McKey D Domestication and diversity in Manioc (*Manihot esculenta *Crantz ssp. *esculenta*, Euphorbiaceae) Current Anthropology 2008 49 1119 1128 10.1086/593119

[B34] Parker KC Hamrick JL Hodgson WC Trapnell DW Parker AJ Kuzoff RK Genetic consequences of pre-columbian cultivation for *Agave murpheyi *and *A. delamateri *(Agavaceae) American Journal of Botany 2007 94 1479 1490 10.3732/ajb.94.9.1479 21636515

[B35] Birmeta G Nybom H Bekele E RAPD analysis of genetic diversity among clones of the Ethiopian crop plant *Ensete ventricosum* Euphytica 2002 124 315 325 10.1023/A:1015733723349

[B36] Jarvis DI Hodgkin T Wild relatives and crop cultivars: detecting natural introgression and farmer selection of new genetic combinations in agroecosystems Molecular Ecology 1999 8 S159 S173 10.1046/j.1365-294X.1999.00799.x

[B37] Leotard G Duputié A Kjellberg F Douzery EJP Debain C De Granville JJ Mckey D Phylogeography and the origin of cassava: new insights from the northern rim of the Amazonian basin Molecular Phylogenetics and Evolution 2009 53 329 334 10.1016/j.ympev.2009.05.003 19435608

[B38] Gleadow RM Edwards EJ Evans JR Changes in nutritional value of cyanogenic Trifolium repens grown at elevated atmospheric CO_2_ Journal of Chemical Ecology 2009 35 476 478 10.1007/s10886-009-9617-5 19352773

[B39] IPGRI, CIHEAM Descriptors for fig 2003 International Plant Genetic Resources Institute, Rome, Italy, and International Centre for Advanced Mediterranean Agronomic Studies, Paris, France 52

[B40] Khadari B Oukabli A Ater M Mamouni A Roger JP Kjellberg F Molecular characterization of Moroccan fig germplasm using Intersimple Sequence Repeat and Simple Sequence Repeat markers to establish a reference collection HortScience 2004 40 29 32

[B41] Tessier C David J This P Boursiquot JM Charrier A Optimization of the choice of molecular markers for varietal identification in *Vitis vinifera *L Theoretical and Applied Genetics 1999 98 171 177 10.1007/s001220051054

[B42] Belkhir K Borsa P Chikhi L Raufaste N Bonhomme F GENETIX 4.05, logiciel sous Windows TM pour la génétique des populations Laboratoire Génome, Populations, Interactions, CNRS UMR 5000, Université de Montpellier II, Montpellier, France 1996

[B43] Nei M Estimation of average heterozygosity and genetic distance from a small number of individuals Genetics 1978 89 583 590 17248844 10.1093/genetics/89.3.583 PMC1213855

[B44] Raymond M Rousset F GENEPOP (version 1.2): population genetics software for exact tests and ecumenicism Journal of Heredity 1995 86 248 249

[B45] Raymond M Rousset F An exact test for population differentiation Evolution 1995 49 1280 1283 10.2307/2410454 28568523

[B46] Hardy O Vekemans X SPAGeDi: a versatile computer program to analyse spatial genetic structure at the individual or population Molecular Ecology Notes 2002 2 618 620 10.1046/j.1471-8286.2002.00305.x

[B47] Loiselle BA Sork VL Nason J Graham C Spatial genetic structure of a tropical understory shrub, *Psychotria officinalis *(Rubiaceae) American Journal of Botany 1995 82 1420 1425 10.2307/2445869

