# Supplementary material for: Traditional agroecosystems as conservatories and incubators of cultivated plant varietal diversity: the case of fig (Ficus carica L.) in Morocco
Source: BMC Plant Biol. 2010 Feb 18;10:28. doi: 10.1186/1471-2229-10-28 (PMC2844065; doi:10.1186/1471-2229-10-28)
Supplement: Additional file 2 — List of groups of closely related genotypes with skin color fruit. This file describes a list of groups of closely related genotypes differed only by 1 to 3 alleles and considered to be somatic variants of a single clone. [file 1471-2229-10-28-S2.DOC]

**Additional File 2. List of groups of closely related genotypes with skin color fruit.**

| **Groups of closely related genotypes** | **Skin color** |
| --- | --- |
| Ikoran Imelalen -IVA1-T1-P001=Ikoran Ihebchan -IVA1-T2-P001^a^ = Mcherta Sghira-IVB1-T9-P001 ≈ Ikoran Imelalen -IVA1-T4-P042 | Green clear |
| Ikoran Imelalen-IVA1-T6-P002 = Ikoran Imelalen-IVA1-T7-P002 =Ikoran Izeghzaouen -IVA2-T1-P002 = Ikoran Ihebchan^a^ -IVA2-T2-P002 = Unnamed-IVA2-T3-P002 ≈ Ikoran Imelalen-IVA1-T5-P043 ≈ Byed Aadi-IVB1-T1-P054 | Green clear |
| Sbelyonia -IVB1-T3-P004 = Biyadi-IVC1-T1-P004 = Biyadi-IVC1-T1P004= Sbelyonia -IVB1-T3-P004 ≈ El Messari-IIB1-T7-P117 ≈ Unnamed-IA2-T4-P016 ≈ El Messari-IID1-T3-P003 ≈ El Messari-IIB1-T7-P117 ≈ Lebyed Lhor-IIB1-T8-P118 ≈ Biyadi-IVC1-T1-P004 | Green clear |
| Rhoudane-IIF1-T12-P006 = Rhoudani-IIIA1-T4-P006 = Taberchant-IIIA2-T2-P006 = Unnamed-IIIB2-T3-P006 = Khel kbira-IVB1-T5-P006 = Kehli-IVC1-T2-P006 = Kehli-IVC1-T4-P006, Kehli Beldi-IVC2-T2-P006 = Kehla Hora-VA2-T6-P006 = El Hemra-VB1-T1-P006 = El Kehla-VB1-T3-P006 = Taroumit (Taberchant)-VB3-T2-P006 = Taroumit-VB3-T3-P006 = El Kehla (Rhoudani)-VC1-T1-P006 = El Kehla-VC1-T4-P006 ≈ Tahadakt-IB1-T3-P036 | Purple black |
| Bida-IIIA1-T3-P007 = Jebli(Tnakssi)-IVC2-T5-P007 = Tabansout-IVE1-T9-P007 = El Modakssi-VB1-T4-P007 ≈ Erkik-IIF1-T10-P149 ≈ Tabnaquest-IVE1-T5-P072 | Green clear |
| Toumlilt-IVD1-T3-P008 = Toumlilt-IVD1-T4-P008 ≈ Takheddout-IVE1-T7-P074 | Green clear |
| Tamellalt-IIIA2-T1-P013 = Tamellalt-IIIA2-T3-P013 ≈ Ouednakssi-IIIA2-T5-P161 | Green clear |
| Khodri-IA1-T2-P014 = Harcha-IA1-T9-P014 = Bouaniyek-IA3-T1-P014 = Lkhila-IB1-T2-P014^a^ = Elharchia-IB1-T4-P014 = Harchi Lebyed-IB2-T1-P014 = Saaidi Lkhel-IB5-T3-P014^a^ = Saaidi Lbyed-IB5-T4-P014 ≈ Unnamed-IIA1-T3-P111 ≈ Elbaghi-IB1-T10-P018^a^ | Green |
| Khelia-IA1-T7-P020^a^ = Lemdar Lbyed-IB3-T2-P020 ≈ Lebyed-IA1-T11-P019 | Green clear |
| Aounq El Hmam-IIE2-T2-P029 = Aryel-IIF2-T3-P029 ≈ Irgui-IIE2-T1-P135 ≈ Irgui-IIE2-T13-P141 | Brown red |
| Hafer El Brhel-IIE2-T3-P030 = Hafer El Brhel-IIE2-T9-P030 ≈ Khehla-IIE1-T1-P129 | Brown red |
| El Qoti-IIB1-T9-P119 ≈ Tayehmet-IB4-T2-P040 | Green yellow |
| Harchi Lebyed-IA2-T9-P170 ≈ Sbaa Orghod-IID1-T7-P104 | Green clear |
| Jarghjogha-IIE2-T10-P138 ≈ Kehla-IIE2-T5-P136 | Black |
| Unnamed-IVA3-T5-P048 ≈ Unnamed-IVA3-T9-P052 ≈ Unnamed-IVA3-T7-P050 | Green clear |
| Tahejajt (Tabekhant)-IVD1-T2-P063 ≈ Taberchant-Tahejajt-IVD1-T7-P066 | Black |
| Tassdjante-VA1-T5-P081 ≈ Unnamed-VA1-T6-P082 | Black |
| Asslia-IA1-T6-P010 ≈ Assal-Fassi-IIC1-T8-P128 | Brown red |
| Rhoudane-IA1-T10-P021 ≈ Rhoudane-IIC1-T6-P028 ≈ Unnamed-IIA1-T2-P110 ≈ Rhoudane-IIE2-T14-P142 | Purple black |
| Rhoudane Khadra-VIA1-T8-P181 ≈ Rhoudania Asslia-VIA1-T9-P182 | Purple black |
| Aïn Hejla-VIA1-T2-P175 ≈ Aïn Hejla-VC1-T3-P173 | Brown red |
| Hambri-IB5-T9-P100 ≈ Sbaa Orghod-IID1-T7-P104 | Green clear |
| Takhelwit (Taberchant)-VB3-T1-P096 ≈ Takhelwit-VB3-T4-P097 | Black |
| Zerki-IID1-T4-P102 ≈ Zrirek-IIB1-T4-P115 ≈ Zrirek-IIB1-T13-P121 ≈ Zerki-IID1-T8-P105 | Brown reddish |

= identical genotypes; ≈ genotypes differing by 1 to 3 alleles; ^a^ varieties differing by fig skin color .
