# Supplementary material for: Traditional agroecosystems as conservatories and incubators of cultivated plant varietal diversity: the case of fig (Ficus carica L.) in Morocco
Source: BMC Plant Biol. 2010 Feb 18;10:28. doi: 10.1186/1471-2229-10-28 (PMC2844065; doi:10.1186/1471-2229-10-28)
Supplement: Additional file 3 — Cases of synonymy. This file describes the cases of synonymy (several variety names for one genotype) observed among cultivated fig trees in Morocco. [file 1471-2229-10-28-S3.DOC]

Additional File 3. Cases of synonymy.

| **Synonymy and varietal identity** | **Number of tree** | **Dissimilar name** |
| --- | --- | --- |
| *Ikoran Imelalen-IVA1-T1-P001, Ikoran Ihebchan-IVA1-T2-P001, Mcherta Sghira-IVB1-T9-P001* | 3 | 3 |
| *Ikoran Imelalen-IVA1-T6-P002, Ikoran Imelalen-IVA1-T7-P002, Ikoran Izeghzaouen -IVA2-T1-P002, Ikoran Ihebchan -IVA2-T2-*P002, *Unnamed-IVA2-T3-P002* | 5 | 3 |
| Bacoura-IB1-T14-P003, Taballacht-IB5-T1-P003, Beret Aïcha-IB5-T6-P003, Johri-IID1-T2-P003, El Messari-IID1-T3-P003, Jaadi-IIF1-T8-P003, Iri O Tbir = Aounq Hmam-IIIB1-T4-P003, Unnamed-IIIB1-T6-P003, Unnamed-IVA3-T4-P003 | 9 | 7 |
| Sbelyonia -IVB1-T3-P004, Biyadi-IVC1-T1-P004 | 2 | 1 |
| Mazouzi (Chetoui)-IA3-T2-P005, Sbelyonia -IVB1-T4-P005, Chetwi Sbelyoni-IVC1-T3-P005, Sbelyoni-IVC2-T4-P005 | 4 | 2 |
| Rhoudane-IIF1-T12-P006, Rhoudani-IIIA1-T4-P006, Taberchant-IIIA2-T2-P006, Unnamed-IIIB2-T3-P006, Khel Kbira-IVB1-T5-P006, Kehli-IVC1-T2-P006, Kehli 2-IVC1-T4-P006, Kehli Beldi-IVC2-T2-P006, Kehla Hora-VA2-T6-P006, El Hemra-VB1-T1-P006, El Kehla-VB1-T3-P006, Taroumit (Taberchant)-VB3-T2-P006, Taroumit-VB3-T3-P006, El Kehla (Rhoudani)-VC1-T1-P006, El Kehla-VC1-T4-P006 | 15 | 7 |
| Bida-IIIA1-T3-P007, Jebli (Tnakssi)-IVC2-T5-P007, Tabansout-IVE1-T9-P007, El Modakssi-VB1-T4-P007 | 4 | 4 |
| Toumlilt-IVD1-T3-P008, Toumlilt-IVD1-T4-P008 | 2 | 1 |
| *Tahjajt (Tabekhant)-IVE1-T2-P009*, *Toumlilt-IVE1-T10-P009* | 2 | 2 |
| Sebtawi-IA1-T1-P010, Assel-IA1-T4-P010, Asslia-IA1-T6-P010, Assal-IID1-T6-P010, Zerka-VA2-T1-P010 | 5 | 3 |
| Kehla Rhoudania-VA2-T2-P011, Kehla-VA2-T8-P011 | 2 | 1 |
| *Lemtel-IIF1-T7-P012*, *Lmetri-IIF1-T16-P012*, *Rhoudane-IIIB1-T3-P012*, *Lendar-IIIB2-T2-P012* | 4 | 4 |
| Tamellalt-IIIA2-T1-P013, Tamellalt-IIIA2-T3-P013 | 2 | 1 |
| *Khodri-IA1-T2-P014*, *Harcha-IA1-T9-P014*, *Bouaniyek-IA3-T1-P014*, *Lkhila-IB1-T2-P014*, *Elharchia-IB1-T4-P014*, *Harchi Lebyed-IB2-T1-P014*, *Saaidi Lkhel-IB5-T3-P014*, *Saaidi Lbyed-IB5-T4-P014* | 8 | 7 |
| Homar -IA2-T1-P015, Hefri-IA2-T7-P015 | 2 | 2 |
| Unnamed -IA2-T4-P016, Beyota-IB1-T8-P016 | 2 | 1 |
| Unnamed-IA2-T5-P017, EL quoti-IA3-T3-P017 | 2 | 1 |
| Kohli-IA2-T8-P018, Elbaghi-IB1-T10-P018 | 2 | 2 |
| *Lebyed-IA1-T11-P019, Bacora-IA3-T5-P019, Abacor Lebyed-IB3-T1-P019, Lemdar-IIC1-T5-P019, Lemdar Lekhel -IID1-T11-P019* | 5 | 5 |
| *Khelia-IA1-T7-P020, Lemdar Lebyed-IB3-T2-P020* | 2 | 2 |
| Rhoudane-IA1-T10-P021, Rhoudane-IB1-T15-P021 | 2 | 1 |
| Elferzaoui-IB1-T5-P022, Taberant-IB5-T2-P022 | 2 | 2 |
| Ghaouzia-IB1-T7-P023, Arifi-IB2-T2-P023, Lwizi-IB4-T1-P023 | 3 | 3 |
| Meltoufa-IB1-T6-P024, Amlatou-IB5-T8-P024 | 2 | 2 |
| Chetwia-IB1-T11-P025, Tazant-IB3-T3-P025 | 2 | 2 |
| Berrani-IIB1-T1-P026, Lemdar-IIB1-T3-P026, Lemdar Lebyed-IIB1-T5-P026 | 3 | 2 |
| El Fassi-IB6-T2-P027, El Fassi-IIB1-T10-P027, El Fassi-IIE2-T6-P027, El Fassi-IIE2-T7-P027, Affassi-IIF1-T4-P027, Fassi-IIF1-T5-P027 | 6 | 1 |
| Rhoudane-IIB1-T11-P028, Rhoudane-IIC1-T6-P028, Rhoudane-IIF1-T1-P028 | 3 | 1 |
| Aounq El Hmam -IIE2-T2-P029, Aryel-IIF2-T3-P029 | 2 | 2 |
| Hafer El Brhel-IIE2-T3-P030, Hafer El Brhel-IIE2-T9-P030 | 2 | 1 |
| Sebti-IIE2-T4-P031, El Harcha-IIF2-T2-P031 | 2 | 2 |
| Unnamed-IIE1-T8-P032, Nabout-IIF2-T5-P032 | 2 | 1 |
| Hmimer-IIF1-T15-P033, Noukali-IIF2-T1-P033 | 2 | 2 |
| *Ikoran Imelalen -IVA2-T4-P034, Ikoran Izeghzaouen-IVA2-T5-P034* | 2 | 2 |

In underlined italic type: varieties differing by fig skin color probably due to somatic mutations.
