# Supplementary material for: Traditional agroecosystems as conservatories and incubators of cultivated plant varietal diversity: the case of fig (Ficus carica L.) in Morocco
Source: BMC Plant Biol. 2010 Feb 18;10:28. doi: 10.1186/1471-2229-10-28 (PMC2844065; doi:10.1186/1471-2229-10-28)
Supplement: Additional file 4 — Cases of homonymy. This file describes the cases of homonymy (several genotypes for one variety name) observed among cultivated fig trees in Morocco. [file 1471-2229-10-28-S4.DOC]

Additional File 4. Cases of homonymy.

| Variety names | **Meaning of name** | **Trees** | **Genotypes** | **Dissimilar allele** |
| --- | --- | --- | --- | --- |
| Abacor Lbyed-IB3-T1-P019^ab^ = Bacora-IA3-T5-P019^b^, Bacoura-IB1-T14-P003 | Breba crop | 3 | 2 | 10 |
| El Fassi-IB6-T2-P027^b^ = El Fassi-IIE2-T7-P027^b^ = Fassi-IIF1-T5-P027^b^ = El Fassi-IIE2-T6-P027^b^ = El Fassi-IIB1-T10-P027^b^ = Affassi-IIF1-T4-P027^ab^  Fassi-IA2-T3-P168^b^  El Fassi-IIIA1-T5-P159 | From Fès city | 8 | 3 | 13 to 16 |
| Aïn Hejla-VC1-T3-P173^b^ ≈ Aïn Hejla-VIA1-T2-P175^b^  Aïn Hejla-VIA1-T1-P174^b^ | Partridge eye | 3 | 3 | 2 to 9 |
| Aounq El Hmam-IIE1-T5-P133^b^, Aounq El Hmam-IIE2-T2-P029^b^, Aounq El Hmam-IID1-T12-P108^b^, Aounq Hmam-IIIA1-T2-P158^b^, Aounq El Hmam-IA3-T4-P076^b^, Aounk El Hmam-VIA2-T1-P183 | Pigeon neck | 6 | 6 | 11 to 20 |
| Aryel-IIF2-T3-P029^ab^ ≈ Irgui-IIE2-T1-P135^ab^ ≈ Irgui-IIE2-T13-P141^ab^ | - | 3 | 2 | 1 to 2 |
| Assel-IA1-T4-P010^b^ = Asslia-IA1-T6-P010^b^ = Assel-IID1-T6-P010^b^ ≈ Assel (Fassi)-IIC1-T8-P128^b^ | Honeyed | 4 | 2 | 2 |
| Ikoran Imelalen-IVA1-T6-P002^ab^ = Ikoran Imelalen-IVA1-T7-P002^ab^ ≈ Ikoran Imelalen-IVA2-T4-P034^ab^ ≈ Byed-IVB1-T1-P054^b^  Tamellalt-IIIA2-T1-P013^ad^ = Tamellalt-IIIA2-T3-P013^ad^  Toumlilt-IVD1-T3-P008^ab^ = Toumlilt-IVD1-T4P008^ab^  Ikoran Imelalen -IVA1-T1-P001^ab^ ≈ Ikoran Imelalen -IVA1-T4-P042^ab^  Toumlilt-IVD1-T5-P064^a^, Toumlilt-IVD1-T4-P008^ab^, Toumlilt-IVD1-T9-P068^a^, Toumlilt-IVE1-T6-P073^a^, Toumlilt-IVE1-T10-P009^a^, Tamellalt-VA1-T3-P079^a^, Ikoran Imellalen-IVA1-T5-P043^ab^, Ikoran Imellalen -IVA3-T1-P045^ab^, Beida-VIA1-T6-P179, Beida -VA2-T5-P089, Beida-VA2-T3-P087, Beida-IIIA1-T3-P007, Beyota-IB1-T8-P016, Beyota-IA2-T2-P167, El Beida-VC1-T2-P172, El Beida-VB2-T2-P095, Lebyed-IA1-T11-P019, Biyadi-IVC1-T1-P004, Lebyed Lhor-IIB1-T8-P118^b^ | White | 28 | 25 | 1 to 22 |
| Boufessase-IIE1-T7-P143^b^, Boufessase-IIE1-T3-P131^b^ | - | 2 | 2 | 12 |
| Chetwi Sbelyoni-IVC1-T3-P005^b^ = Mazouzi (Chetwi)-IA3-T2-P005^b^  Chetwia-IVB1-T2-P055, Chetwia-IIIB2-T1-P156, Chetwia-IB1-T11-P025 | Tardy | 5 | 4 | 5 to 17 |
| Harchi Lebyed-IB2-T1-P014^b^ = Harcha-IA1-T9-P014^b^ = El Herchia-IB1-T4-P014^b^  El Hercha-IIF2-T2-P031^b^, El Hercha-IIE2-T11-P139^b^  El Harchi-IIB1-T6-P116, Harchi Lebyed-IA2-T9-P170, Lehrech-VIC1-T1-P189 | Rough | 8 | 6 | 8 to 19 |
| El Hemra-VB1-T1-P006^b^, El Hemra-VB1-T5-P093^b^, El Hemra chetwiya-VA2-T4-P088^b^,  Hmeri-IIC1-T1-P123, Hmimer-IIF1-T15-P033,  Homar-IA1-T3-P163, Homar -IA2-T1-P015  Homri-IIF1-T14-P152 | Red | 8 | 8 | 6 to 19 |
| El Kehla-VC1-T4-P006 ^b^ = El Kehla-VB1-T3-P006 ^b^ = El Kehla (Rhoudani)-VC1-T1-P006 ^b^ = Kehla Hora-VA2-T6-P006 ^b^ = Khel Kbira-IVB1-T5-P006 ^b^ = Kehli Beldi-IVC2-T2-P006 ^b^ = Kehli-IVC1-T4-P006 ^b^ = Kehli-IVC1-T2-P006 ^b^ = Taberchant-IIIA2-T2-P006 ^ab^  Ikoran Ihebchan -IVA1-T2-P001^a^, Ikoran Ihebchan-IVA2-T2-P002 ^ab^, Ikoran Ihebchan -IVA1-T3-P041^ab^, Ikoran Ihebchan-IVA2-T6-P044^ab^, Kehla-IVB1-T7-P057,  Kehla Rhoudania-VA2-T2-P011, Kehla-VA2-T8-P011  Khehla-IIE1-T1-P129, Kehla-IIE2-T5-P136,  Khelia-IA1-T7-P020, Khila-IB1-T2-P014, Kohli-IA2-T8-P018  Kehla-IVB1-T8-P058, Kehla-VIA1-T3-P176, Lkhel-IVC2-T3-P061, Kehla-VIA1-T4-P177, Kehla Hercha-VIA1-T7-P180  Taberchant(Tahejajt)-IVD1-T7-P066^ab^, Taberchante-VA1-T1-P077^ab^, Taberchant-VA1-T10-P086^ab^ | Black | 29 | 20 | 4 to 23 |
| El Messari-IIB1-T7-P117^b^ ≈ El Messari-IID1-T3-P003^b^ | - | 2 | 2 | 1 |
| EL Qouti-IA3-T3-P017, El Qouti-IIB1-T9-P119 | - | 2 | 2 | 16 |
| Erqiq-IIF1-T10-P149, Rqiq-IVD1-T6-P065, Rqiq-IVD1-T8-P067 | Slim | 3 | 3 | 14 to 16 |
| Ghaouzia-IB1-T7-P023^b^ = Lwizi-IB4-T1-P023^b^, Lwizi-IIC1-T2-P124 | Almond | 3 | 2 | 13 |
| Hafer El Brhel-IIE2-T3-P030, Hafer El Brhel-IIE2-T9-P030, Hafer El Brhel-IB3-T4-P166 | Mule hoof | 3 | 2 | 9 |
| Hejaja-IID1-T9-P106, Tahejajt(Tabekhant)-IVD1-T2-P063^ab^, Tahejajt(Tabekhant)-IVE1-T2-P009^ab^ | Black | 3 | 3 | 9 to 16 |
| Ikoran Izeghzaouen-IVA2-T1-P002^ab^ ≈ Ikoran Izeghzaouen -IVA2-T5-P034^ab^  Tazeghzaout-IIIA2-T4-P160^ab^, Tazeghzaout-IIIA2-T6-P162^ab^, Tazeghzaout-IIIB1-T2-P154^ab^, Tazeghzaout-IVE1-T1-P069^a^, Tazeghzaout-VIB1-T2-P186^a^, Tazeghzaout-VA1-T4-P080^a^  Khodri-IA1-T2-P014, Khdaria-VIA1-T5-P178 | Green | 10 | 10 | 1 to 20 |
| *Lemdar Lekhel-IID1-T11P019* = *Lemdar-IIC1-T5P019*^b^ ≈ *Lemdar Lbyed-IB3-T2P020*^b^  Lemdar-IIB1-T3P026^b^ = Lemdar Lbyed-IIB1-T5P026^b^  Lemdar-IIIB2-T2P012 | - | 6 | 4 | 1 to 11 |
| Lmcherta-VB1-T2-P092, Mcherta Sghira-IVB1-T9-P001 | Striped | 2 | 2 | 10 |
| Nabout-IIB1-T12-P120, Nabout Lkhel-IIB1-T2-P114, Nabout-IB1-T12-P038, Nabout-IIF2-T5-P032 | From seedling | 4 | 4 | 12 to 15 |
| Ournakssi-IIF1-T13-P151, Ouednakssi-IIIA1-T1-P157, Ouednakssi-IIIA2-T5-P161, El Modakssi-VB1-T4-P007 | - | 4 | 4 | 10 to 17 |
| Rhoudane-IIB1-T11-P028^b^ = Rhoudane-IIF1-T1-P028^b^ = Rhoudane-IIC1-T6-P028^b^ ≈ Rhoudane-IIE2-T14-P142^b^ ≈ Rhoudane-IA1-T10-P021^b^ ≈ Rhoudane-IB1-T15-P021^b^  Rhoudani-IIIA1-T4-P006 =Rhoudane-IIF1-T12-P006^b^, Rhoudane-IIIB1-T3-P012^b^, Rhoudane-IIF1-T11-P150^b^  Rhoudania Asslia-VIA1-T9-P182^b^ ≈ Rhoudane Khedra-VIA1-T8-P181^b^ | - | 12 | 8 | 1 to 20 |
| Sbelyoni-IVC2-T4-P005^b^=Sbelyonia -IVB1-T4-P005^b^, Sbelyonia -IVB1-T3-P004^b^ | From Spain | 3 | 2 | 12 |
| Sebtawi-IA1-T1-P010, Sebti-IIE2-T4-P031^b^, Sebti-IIF1-T6-P146^b^ | - | 3 | 2 | 13 to 15 |
| Taberrant-IB5-T2-P022^a^, Berrani-IIB1-T1-P026 | Foreign | 2 | 2 | 10 |
| Taskourit-VIC1-T5-P193^a^, Taskourt-IVE1-T8-P075^a^ | Small partridge | 2 | 2 | 15 |
| Zerki-IID1-T4-P102^b^ ≈ Zrirek-IIB1-T4-P115^b^ ≈ Zrirek-IIB1-T13-P121^b^ ≈ Zerki-IID1-T8-P105^b^  Zerka-VA2-T1-P010^b^, Zerka-VA2-T9-P091^b^ | Blue | 6 | 6 | 2 to 13 |
| Takhelwit-VB3-T4-P097^ab^ ≈ Takhelwit (Taberchant)-VB3-T1-P096^ab^ | Wild | 2 | 2 | 3 |
| Tounghalt-VIB1-T1-P185^ab^, Tounghalt-VIB1-T3-P187^ab^ | - | 2 | 2 | 18 |

Genotypes linked with the sign ≈ only differ by 1-3 alleles and are therefore considered to be somatic variants of a single clone; in underlined italic type: genotypes presenting fig skin color variants probably due to somatic mutations; ^a^ Berber variety name; ^b^ varieties presenting similar pomological traits.
